# Supplementary material for: Anti-Inflammatory, Antidiabetic Properties and In Silico Modeling of Cucurbitane-Type Triterpene Glycosides from Fruits of an Indian Cultivar of Momordica charantia L
Source: Molecules. 2021 Feb 16;26(4):1038. doi: 10.3390/molecules26041038 (PMC7920048; doi:10.3390/molecules26041038)
Supplement: Supplementary file 1 [file molecules-26-01038-s001.zip › molecules-1097994-SI.pptx]

## Slide 1
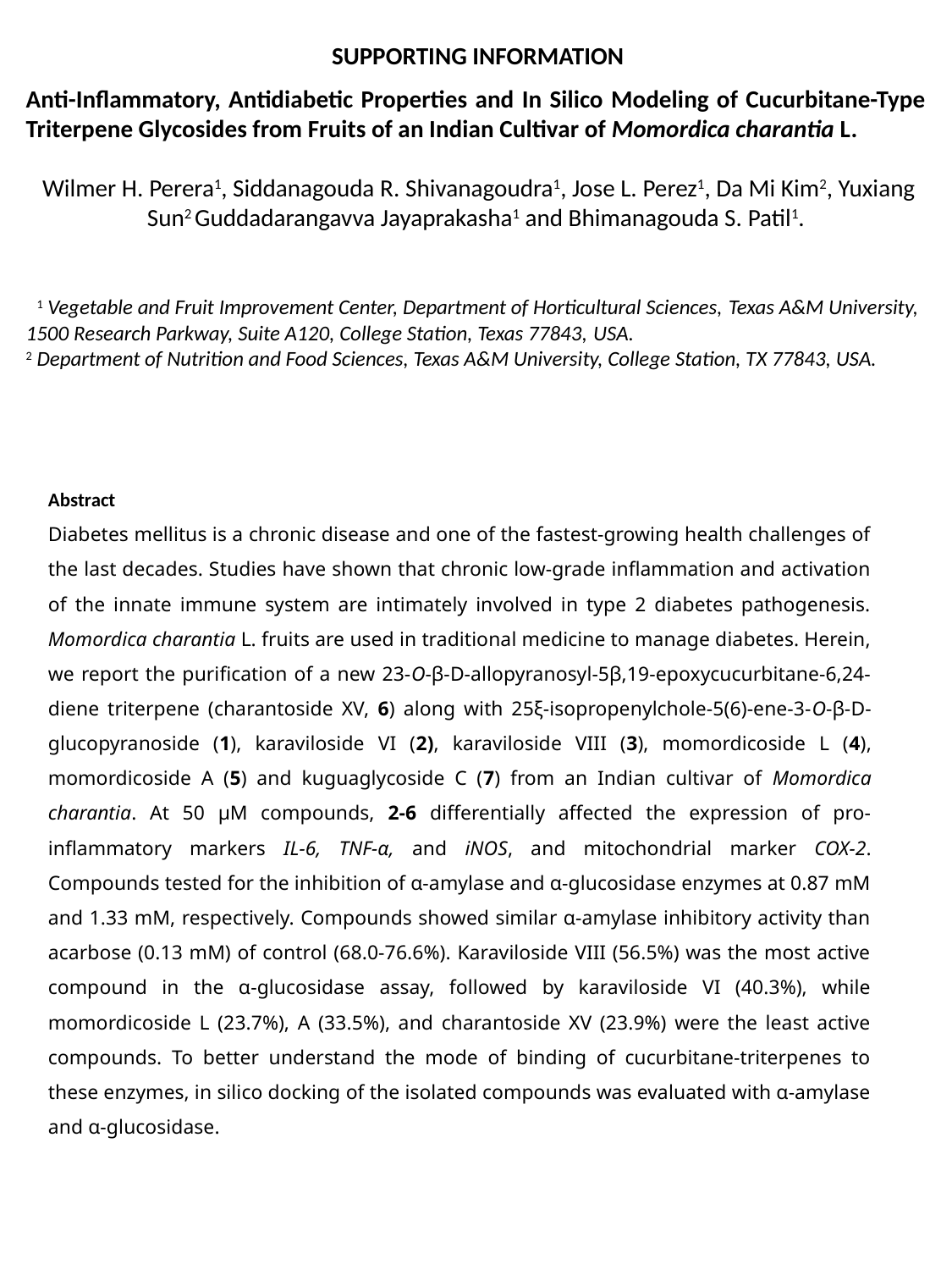

SUPPORTING INFORMATION
Anti-Inflammatory, Antidiabetic Properties and In Silico Modeling of Cucurbitane-Type Triterpene Glycosides from Fruits of an Indian Cultivar of Momordica charantia L.
 Wilmer H. Perera1, Siddanagouda R. Shivanagoudra1, Jose L. Perez1, Da Mi Kim2, Yuxiang Sun2 Guddadarangavva Jayaprakasha1 and Bhimanagouda S. Patil1.
  1 Vegetable and Fruit Improvement Center, Department of Horticultural Sciences, Texas A&M University, 1500 Research Parkway, Suite A120, College Station, Texas 77843, USA.
2 Department of Nutrition and Food Sciences, Texas A&M University, College Station, TX 77843, USA.
Abstract
Diabetes mellitus is a chronic disease and one of the fastest-growing health challenges of the last decades. Studies have shown that chronic low-grade inflammation and activation of the innate immune system are intimately involved in type 2 diabetes pathogenesis. Momordica charantia L. fruits are used in traditional medicine to manage diabetes. Herein, we report the purification of a new 23-O-β-d-allopyranosyl-5β,19-epoxycucurbitane-6,24-diene triterpene (charantoside XV, 6) along with 25ξ-isopropenylchole-5(6)-ene-3-O-β-d-glucopyranoside (1), karaviloside VI (2), karaviloside VIII (3), momordicoside L (4), momordicoside A (5) and kuguaglycoside C (7) from an Indian cultivar of Momordica charantia. At 50 µM compounds, 2-6 differentially affected the expression of pro-inflammatory markers IL-6, TNF-α, and iNOS, and mitochondrial marker COX-2. Compounds tested for the inhibition of α-amylase and α-glucosidase enzymes at 0.87 mM and 1.33 mM, respectively. Compounds showed similar α-amylase inhibitory activity than acarbose (0.13 mM) of control (68.0-76.6%). Karaviloside VIII (56.5%) was the most active compound in the α-glucosidase assay, followed by karaviloside VI (40.3%), while momordicoside L (23.7%), A (33.5%), and charantoside XV (23.9%) were the least active compounds. To better understand the mode of binding of cucurbitane-triterpenes to these enzymes, in silico docking of the isolated compounds was evaluated with α-amylase and α-glucosidase.

## Slide 2
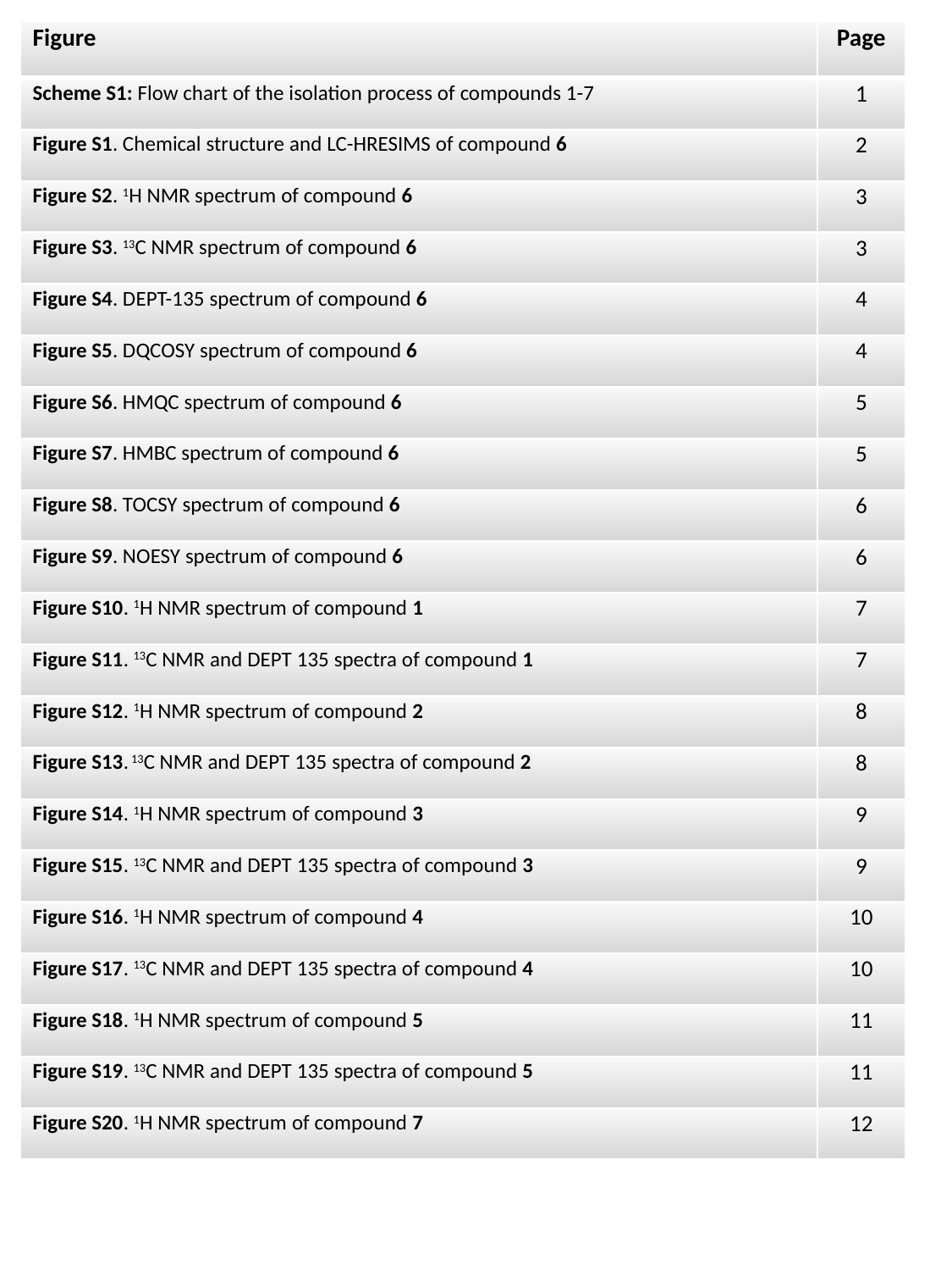

| Figure | Page |
| --- | --- |
| Scheme S1: Flow chart of the isolation process of compounds 1-7 | 1 |
| Figure S1. Chemical structure and LC-HRESIMS of compound 6 | 2 |
| Figure S2. 1H NMR spectrum of compound 6 | 3 |
| Figure S3. 13C NMR spectrum of compound 6 | 3 |
| Figure S4. DEPT-135 spectrum of compound 6 | 4 |
| Figure S5. DQCOSY spectrum of compound 6 | 4 |
| Figure S6. HMQC spectrum of compound 6 | 5 |
| Figure S7. HMBC spectrum of compound 6 | 5 |
| Figure S8. TOCSY spectrum of compound 6 | 6 |
| Figure S9. NOESY spectrum of compound 6 | 6 |
| Figure S10. 1H NMR spectrum of compound 1 | 7 |
| Figure S11. 13C NMR and DEPT 135 spectra of compound 1 | 7 |
| Figure S12. 1H NMR spectrum of compound 2 | 8 |
| Figure S13. 13C NMR and DEPT 135 spectra of compound 2 | 8 |
| Figure S14. 1H NMR spectrum of compound 3 | 9 |
| Figure S15. 13C NMR and DEPT 135 spectra of compound 3 | 9 |
| Figure S16. 1H NMR spectrum of compound 4 | 10 |
| Figure S17. 13C NMR and DEPT 135 spectra of compound 4 | 10 |
| Figure S18. 1H NMR spectrum of compound 5 | 11 |
| Figure S19. 13C NMR and DEPT 135 spectra of compound 5 | 11 |
| Figure S20. 1H NMR spectrum of compound 7 | 12 |

## Slide 3
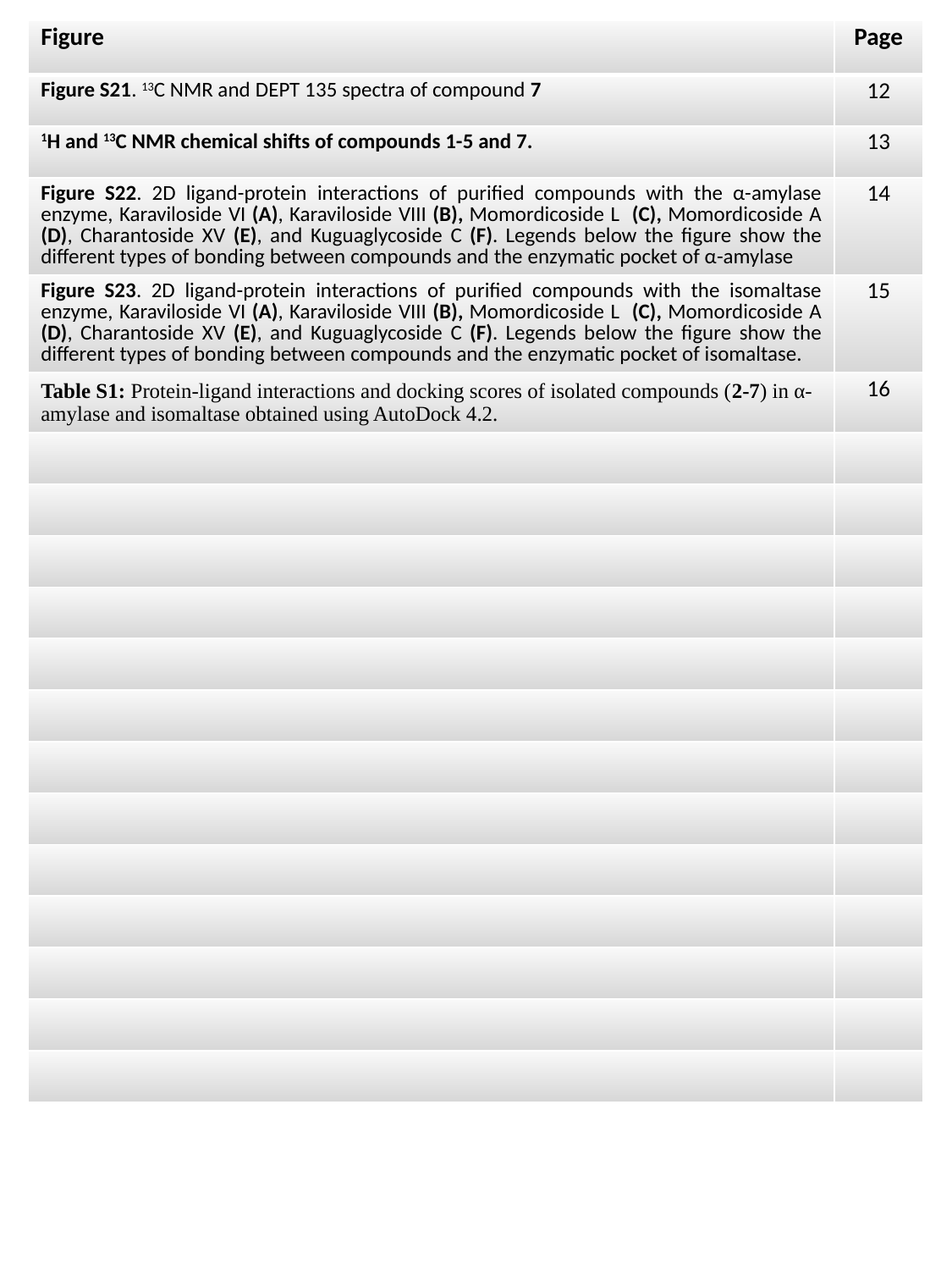

| Figure | Page |
| --- | --- |
| Figure S21. 13C NMR and DEPT 135 spectra of compound 7 | 12 |
| 1H and 13C NMR chemical shifts of compounds 1-5 and 7. | 13 |
| Figure S22. 2D ligand-protein interactions of purified compounds with the α-amylase enzyme, Karaviloside VI (A), Karaviloside VIII (B), Momordicoside L (C), Momordicoside A (D), Charantoside XV (E), and Kuguaglycoside C (F). Legends below the figure show the different types of bonding between compounds and the enzymatic pocket of α-amylase | 14 |
| Figure S23. 2D ligand-protein interactions of purified compounds with the isomaltase enzyme, Karaviloside VI (A), Karaviloside VIII (B), Momordicoside L (C), Momordicoside A (D), Charantoside XV (E), and Kuguaglycoside C (F). Legends below the figure show the different types of bonding between compounds and the enzymatic pocket of isomaltase. | 15 |
| Table S1: Protein-ligand interactions and docking scores of isolated compounds (2-7) in α-amylase and isomaltase obtained using AutoDock 4.2. | 16 |
| | |
| | |
| | |
| | |
| | |
| | |
| | |
| | |
| | |
| | |
| | |
| | |
| | |

## Slide 4
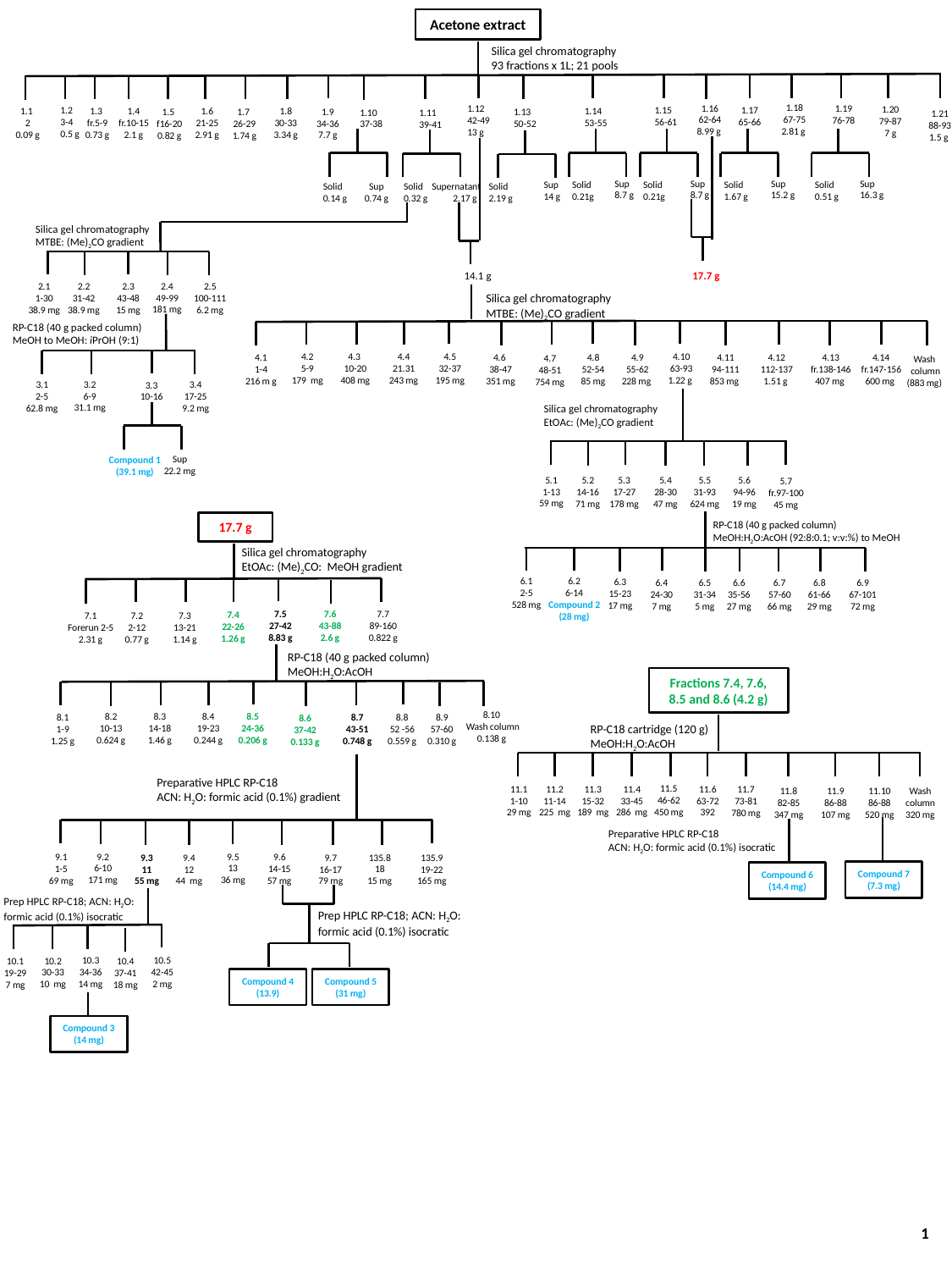

Acetone extract
Silica gel chromatography
93 fractions x 1L; 21 pools
1.18
67-75
2.81 g
1.15
56-61
Sup
8.7 g
Solid
0.21g
1.17
65-66
Sup
15.2 g
Solid
1.67 g
1.19
76-78
Sup
16.3 g
Solid
0.51 g
1.20
79-87
7 g
1.12
42-49
13 g
14.1 g
1.9
34-36
7.7 g
1.14
53-55
Sup
8.7 g
Solid
0.21g
1.4
fr.10-15
2.1 g
1.16
62-64
8.99 g
1.7
26-29
1.74 g
1.8
30-33
3.34 g
1.11
39-41
Supernatant
 2.17 g
Solid
0.32 g
1.2
3-4
0.5 g
1.3
fr.5-9
0.73 g
1.1
2
0.09 g
1.6
21-25
2.91 g
1.13
50-52
Sup
14 g
Solid
2.19 g
1.5
f16-20
0.82 g
1.10
37-38
1.21
88-93
1.5 g
Sup
0.74 g
Solid
0.14 g
17.7 g
Silica gel chromatography
MTBE: (Me)2CO gradient
2.4
49-99
181 mg
2.2
31-42
38.9 mg
2.1
1-30
38.9 mg
2.3
43-48
15 mg
2.5
100-111
6.2 mg
Silica gel chromatography
MTBE: (Me)2CO gradient
RP-C18 (40 g packed column)
MeOH to MeOH: iPrOH (9:1)
4.8
52-54
85 mg
4.5
32-37
195 mg
4.10
63-93
1.22 g
4.3
10-20
408 mg
4.7
48-51
754 mg
4.4
21.31
243 mg
4.12
112-137
1.51 g
4.2
5-9
179 mg
4.14
fr.147-156
600 mg
4.9
55-62
228 mg
Wash
column
(883 mg)
4.13
fr.138-146
407 mg
4.11
94-111
853 mg
4.1
1-4
216 m g
4.6
38-47
351 mg
3.3
10-16
Sup
22.2 mg
Compound 1
(39.1 mg)
3.1
2-5
62.8 mg
3.2
6-9
31.1 mg
3.4
17-25
9.2 mg
Silica gel chromatography
EtOAc: (Me)2CO gradient
5.1
1-13
59 mg
5.2
14-16
71 mg
5.7
fr.97-100
45 mg
5.6
94-96
19 mg
5.4
28-30
47 mg
5.3
17-27
178 mg
5.5
31-93
624 mg
RP-C18 (40 g packed column)
MeOH:H2O:AcOH (92:8:0.1; v:v:%) to MeOH
6.1
2-5
528 mg
6.2
6-14
Compound 2
(28 mg)
6.3
15-23
17 mg
6.6
35-56
27 mg
6.9
67-101
72 mg
6.8
61-66
29 mg
6.5
31-34
5 mg
6.4
24-30
7 mg
6.7
57-60
66 mg
17.7 g
Silica gel chromatography
EtOAc: (Me)2CO: MeOH gradient
7.5
27-42
8.83 g
7.3
13-21
1.14 g
7.7
89-160
0.822 g
7.4
22-26
1.26 g
7.2
2-12
0.77 g
7.1
Forerun 2-5
2.31 g
7.6
43-88
2.6 g
RP-C18 (40 g packed column)
MeOH:H2O:AcOH
Fractions 7.4, 7.6, 8.5 and 8.6 (4.2 g)
8.8
52 -56
0.559 g
8.5
24-36
0.206 g
8.1
1-9
1.25 g
8.3
14-18
1.46 g
8.4
19-23
0.244 g
8.7
43-51
0.748 g
8.2
10-13
0.624 g
8.9
57-60
0.310 g
8.6
37-42
0.133 g
8.10
 Wash column
0.138 g
RP-C18 cartridge (120 g)
MeOH:H2O:AcOH
11.5
46-62
450 mg
11.7
73-81
780 mg
11.2
11-14
225 mg
11.3
15-32
189 mg
11.6
63-72
392
Wash column
320 mg
11.8
82-85
347 mg
11.9
86-88
107 mg
11.10
86-88
520 mg
11.4
33-45
286 mg
11.1
1-10
29 mg
Preparative HPLC RP-C18
ACN: H2O: formic acid (0.1%) gradient
135.9
19-22
165 mg
9.1
1-5
69 mg
9.2
6-10
171 mg
9.5
13
36 mg
Preparative HPLC RP-C18
ACN: H2O: formic acid (0.1%) isocratic
9.6
14-15
57 mg
9.3
11
55 mg
135.8
18
15 mg
9.7
16-17
79 mg
9.4
12
44 mg
Compound 7
(7.3 mg)
Compound 6
(14.4 mg)
Prep HPLC RP-C18; ACN: H2O: formic acid (0.1%) isocratic
Prep HPLC RP-C18; ACN: H2O: formic acid (0.1%) isocratic
Compound 5
(31 mg)
10.5
42-45
2 mg
10.2
30-33
10 mg
10.3
34-36
14 mg
10.1
19-29
7 mg
10.4
37-41
18 mg
Compound 4
(13.9)
Compound 3
(14 mg)
1
4

## Slide 5
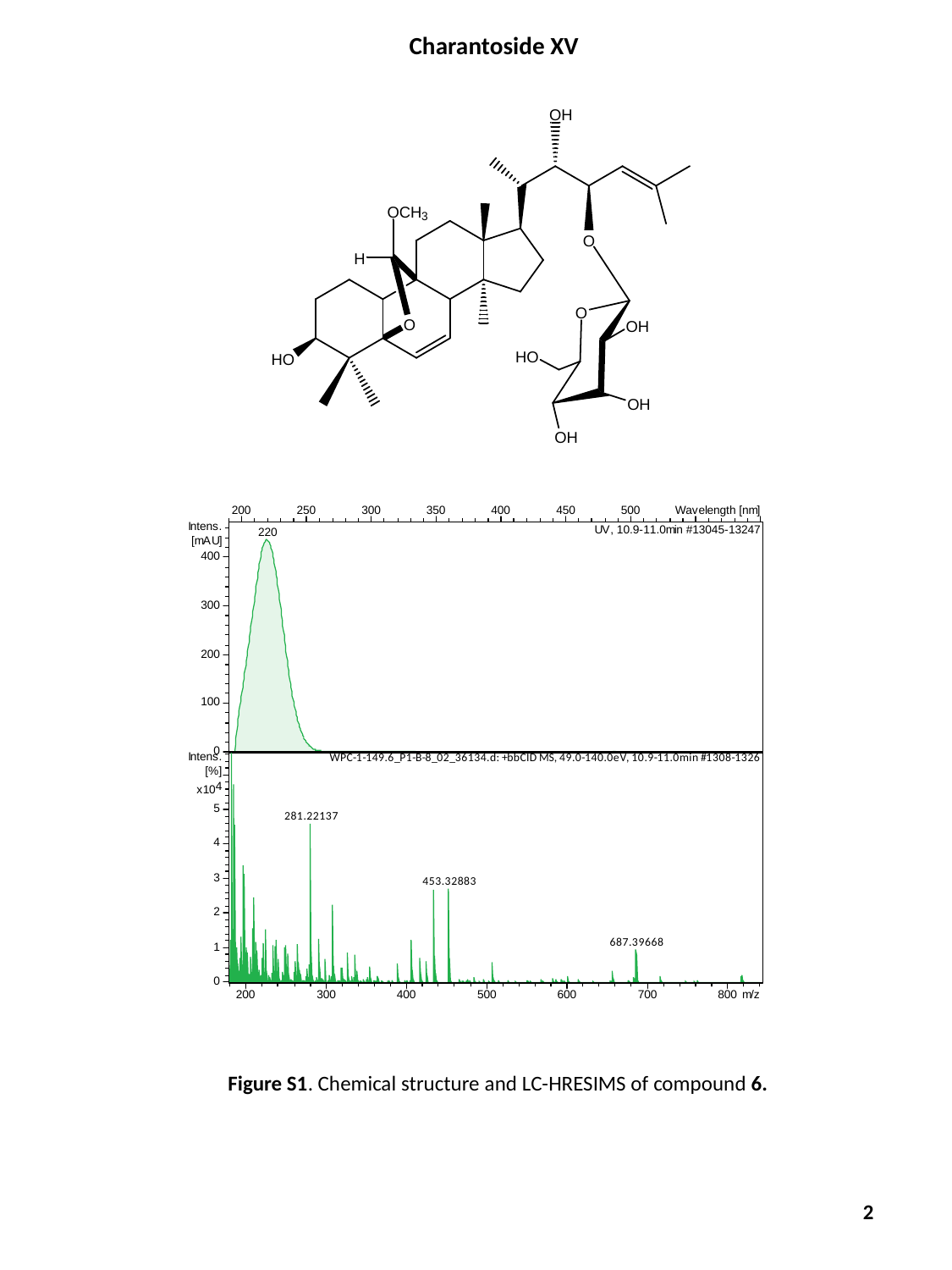

Charantoside XV
Figure S1. Chemical structure and LC-HRESIMS of compound 6.
2

## Slide 6
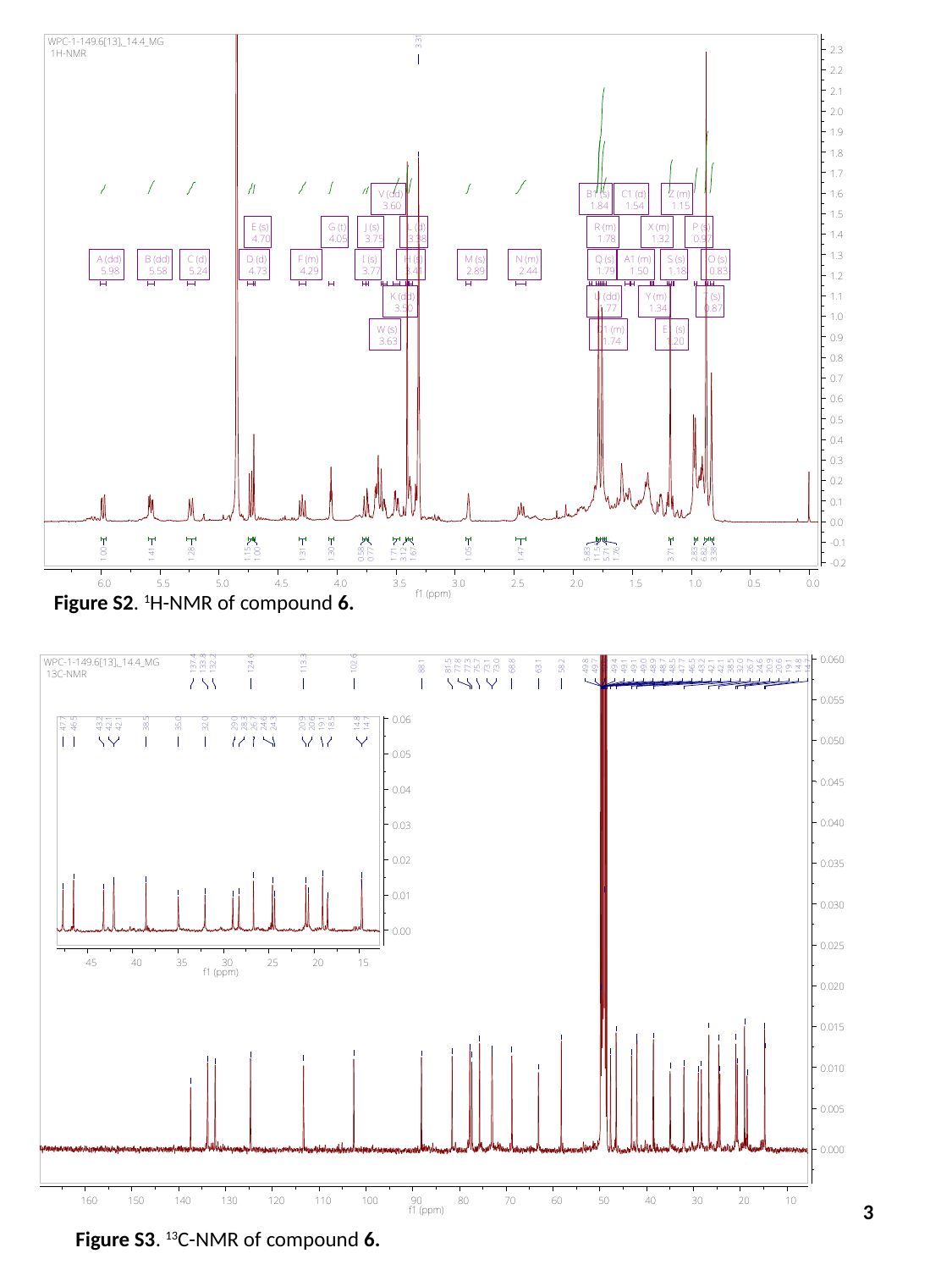

Figure S2. 1H-NMR of compound 6.
3
Figure S3. 13C-NMR of compound 6.

## Slide 7
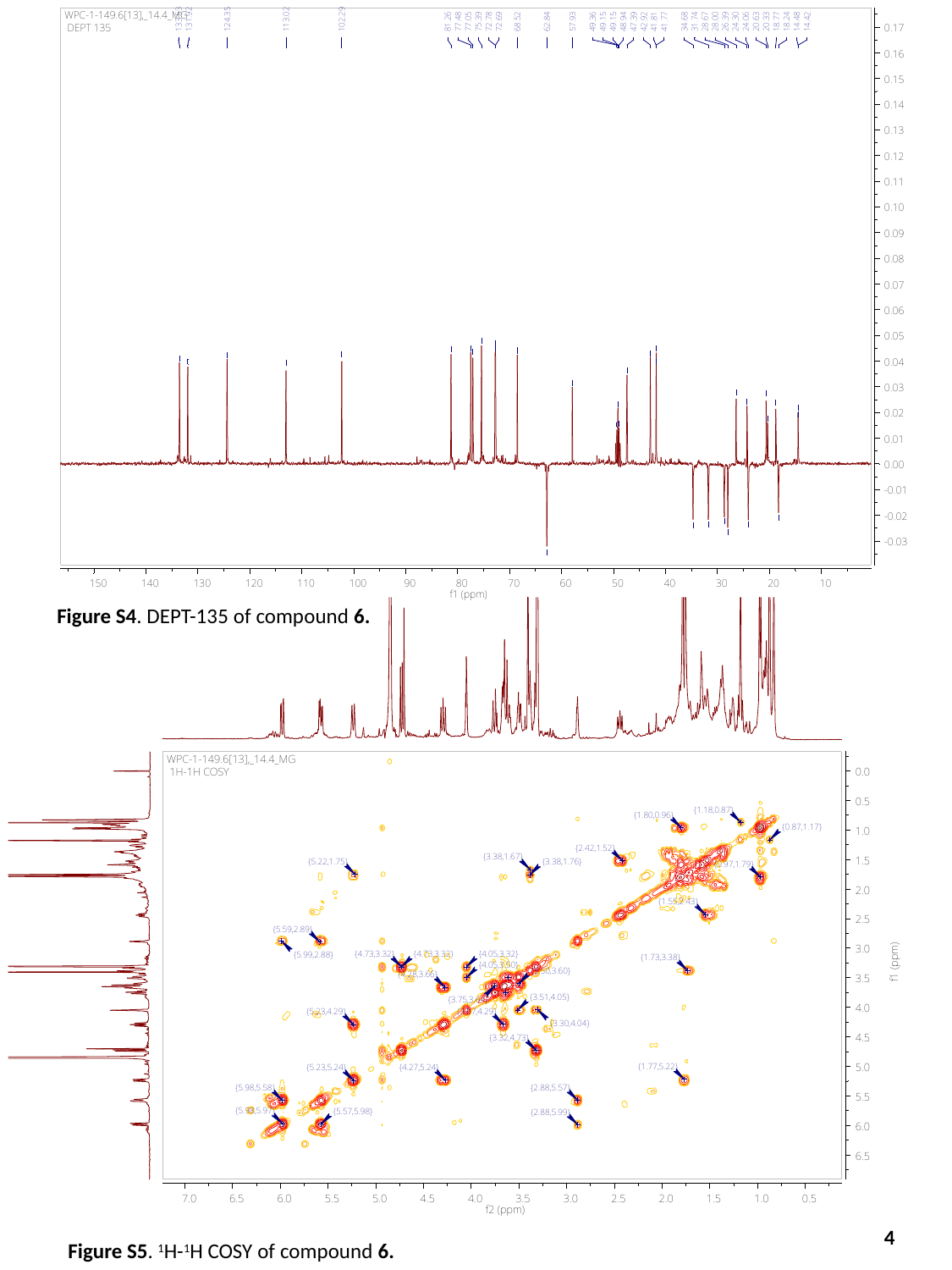

Figure S4. DEPT-135 of compound 6.
4
Figure S5. 1H-1H COSY of compound 6.

## Slide 8
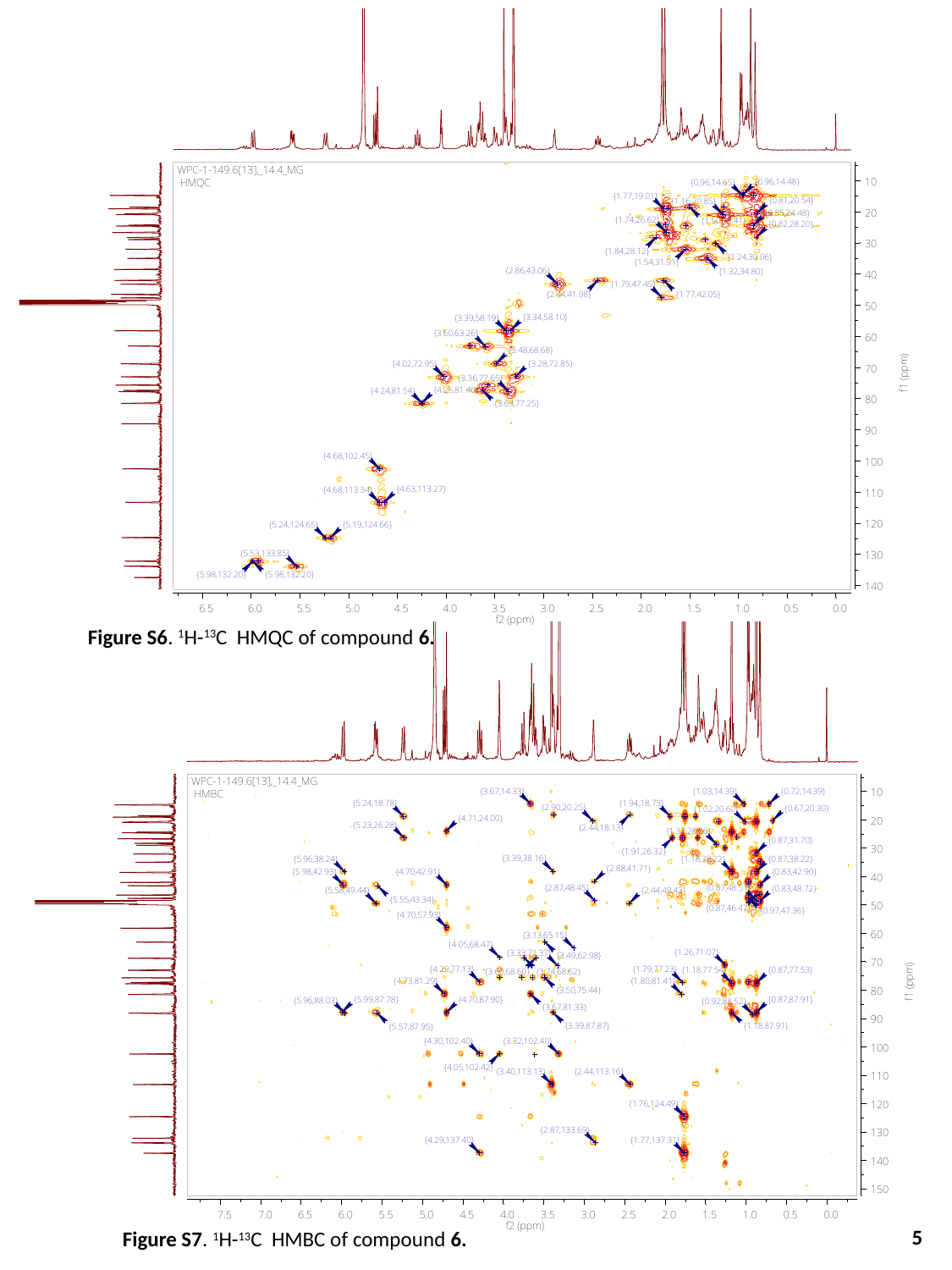

Figure S6. 1H-13C HMQC of compound 6.
5
Figure S7. 1H-13C HMBC of compound 6.

## Slide 9
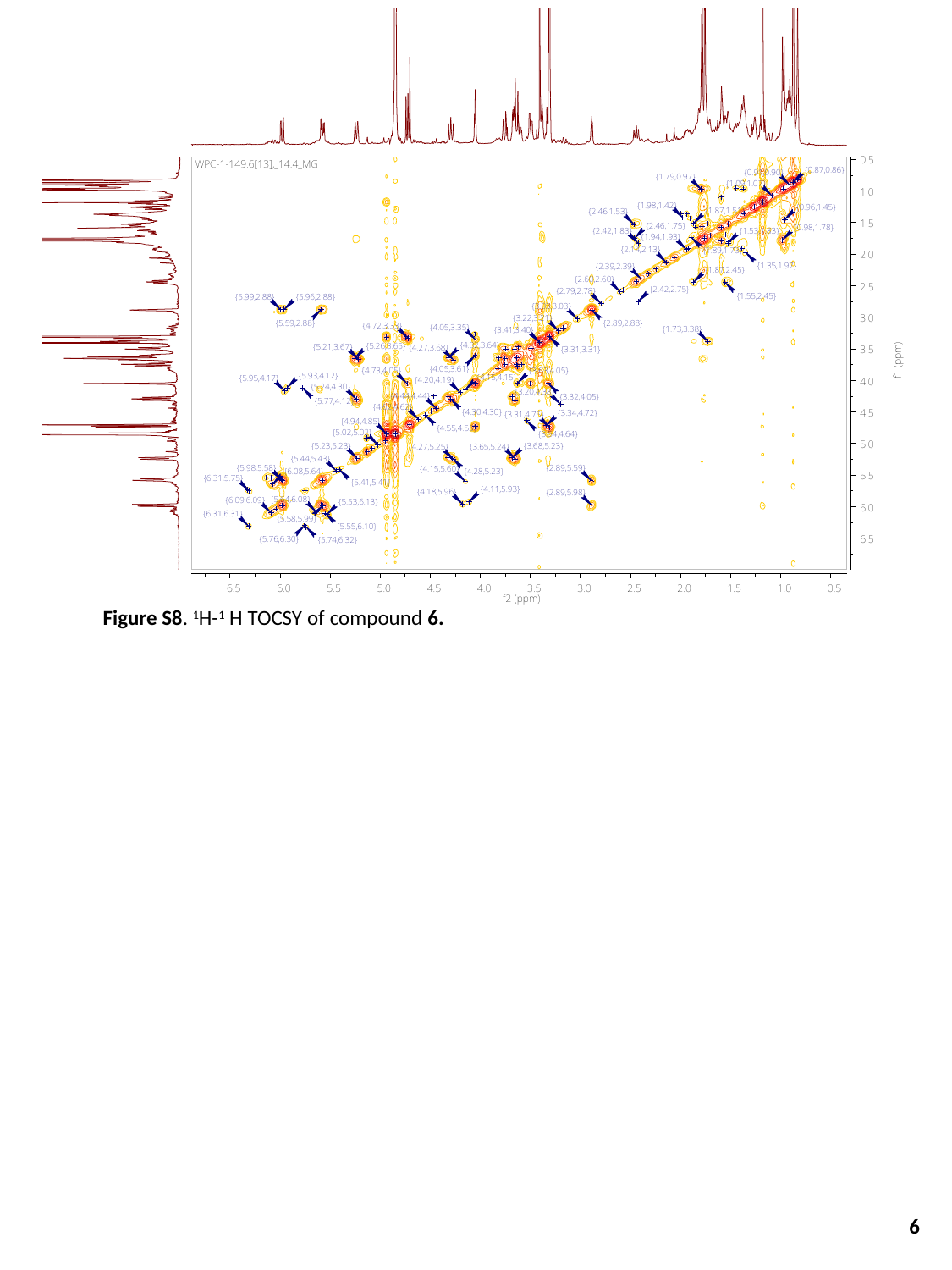

Figure S8. 1H-1 H TOCSY of compound 6.
6

## Slide 10
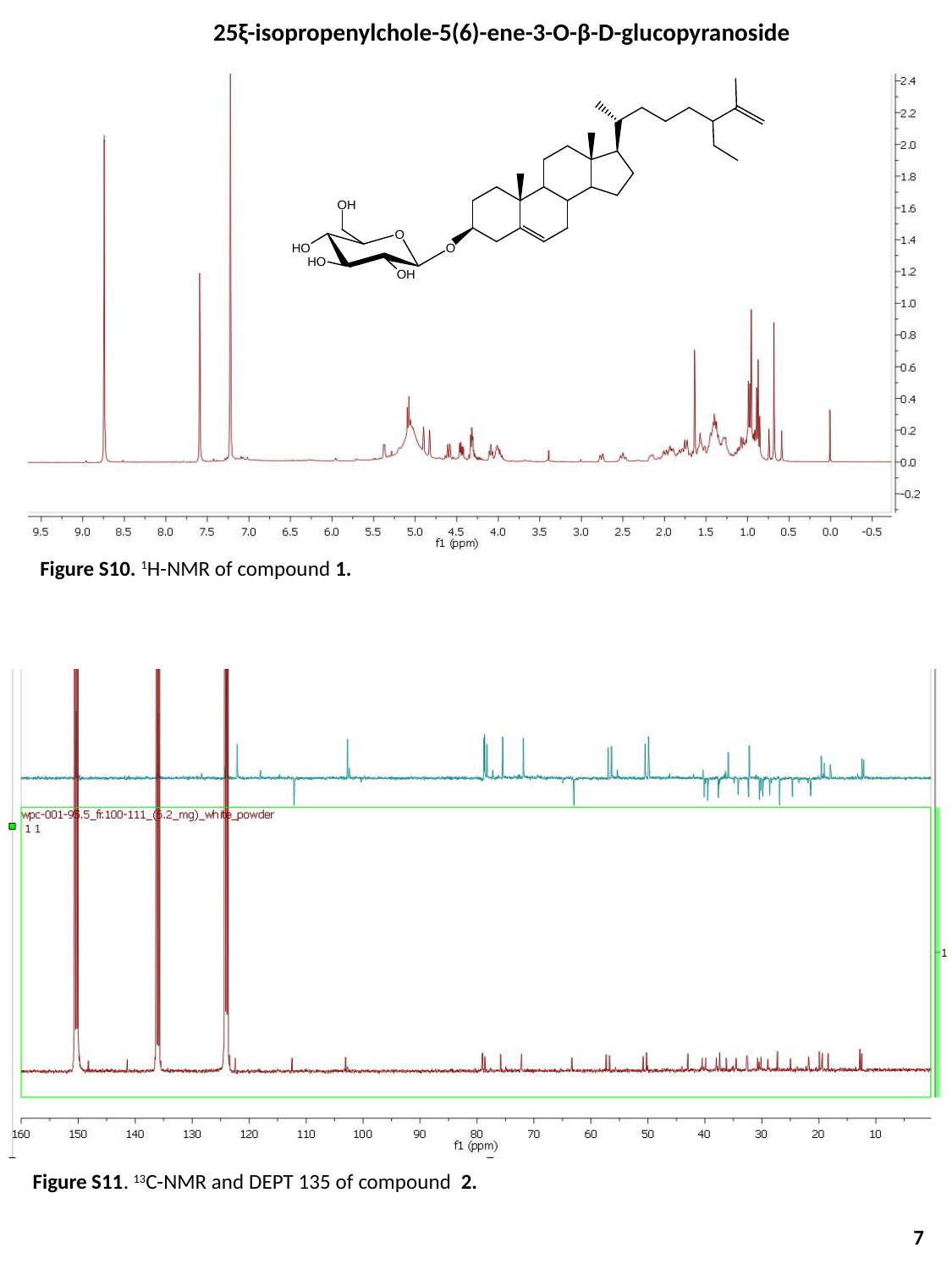

25ξ-isopropenylchole-5(6)-ene-3-O-β-D-glucopyranoside
Figure S10. 1H-NMR of compound 1.
Figure S11. 13C-NMR and DEPT 135 of compound 2.
7

## Slide 11
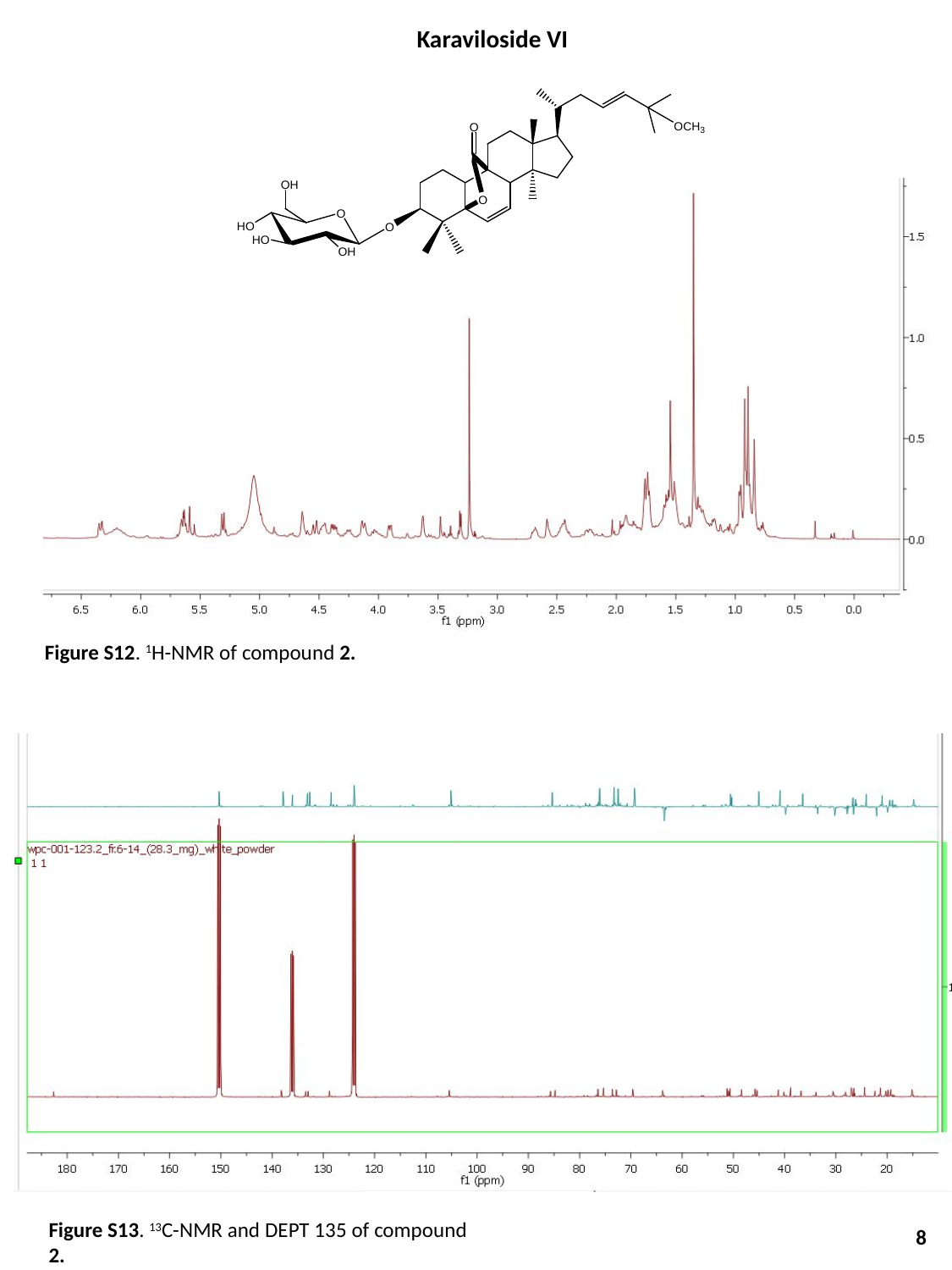

Karaviloside VI
Figure S12. 1H-NMR of compound 2.
8
Figure S13. 13C-NMR and DEPT 135 of compound 2.

## Slide 12
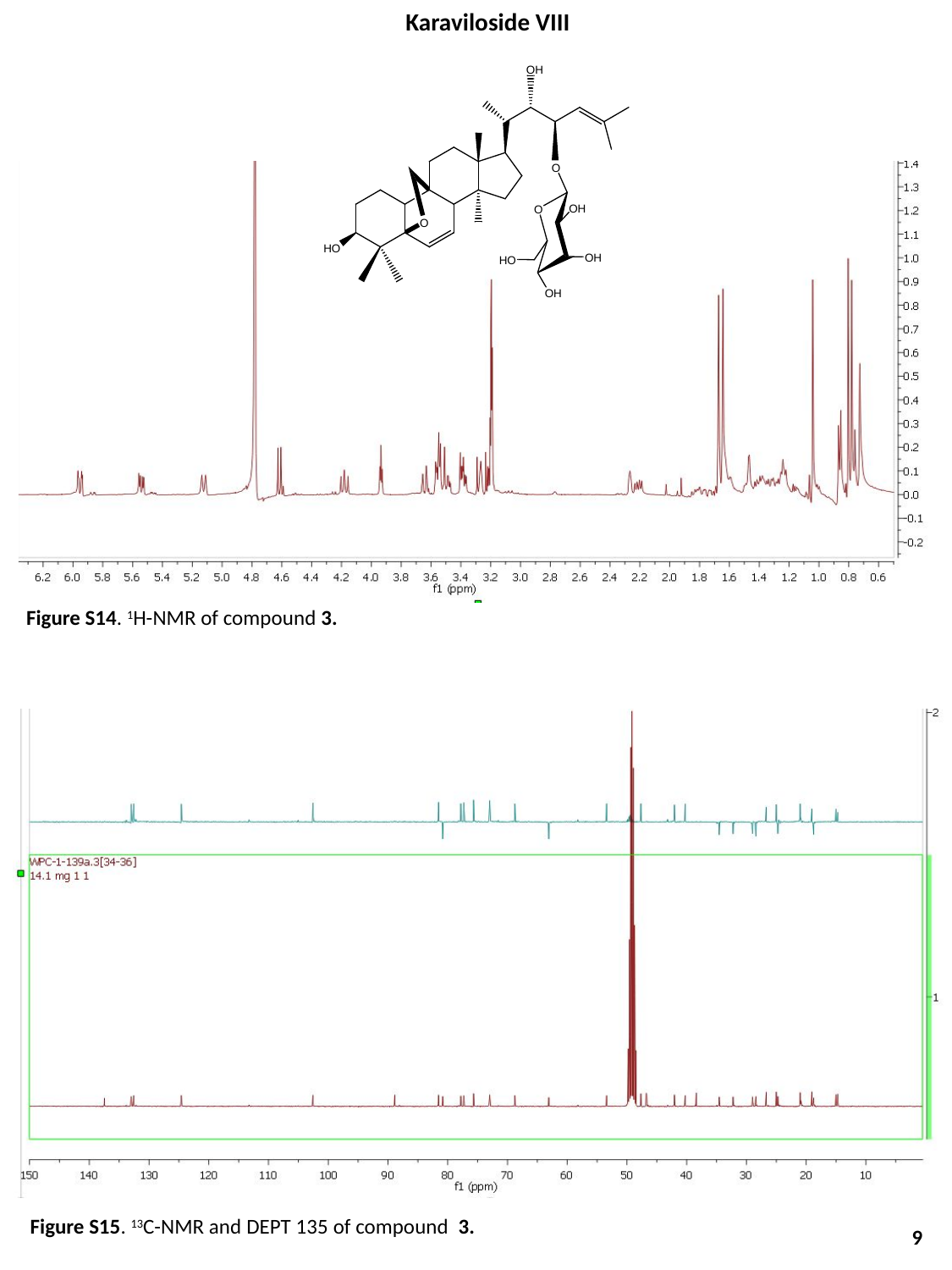

Karaviloside VIII
Figure S14. 1H-NMR of compound 3.
9
Figure S15. 13C-NMR and DEPT 135 of compound 3.

## Slide 13
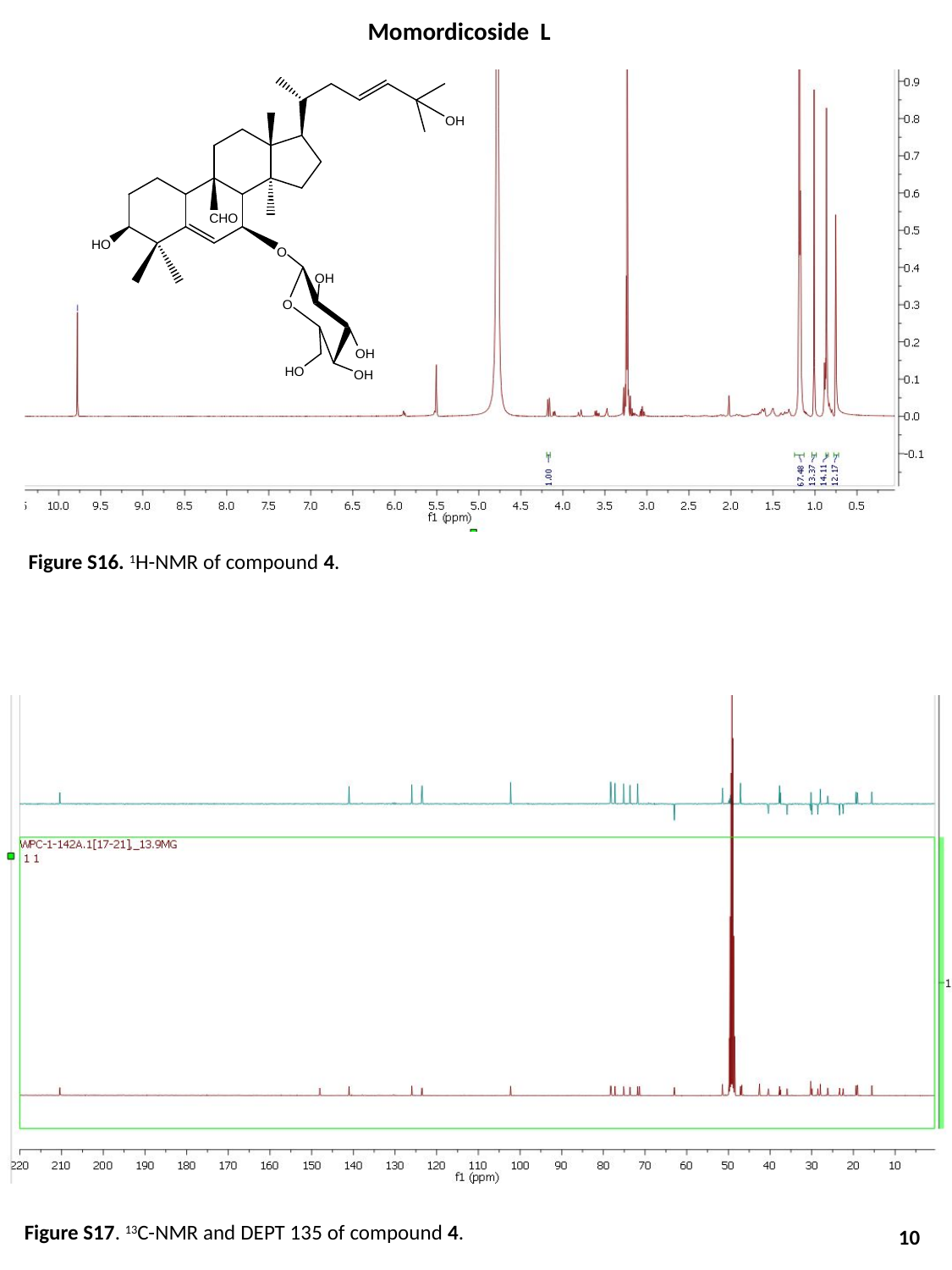

Momordicoside L
Figure S16. 1H-NMR of compound 4.
10
Figure S17. 13C-NMR and DEPT 135 of compound 4.

## Slide 14
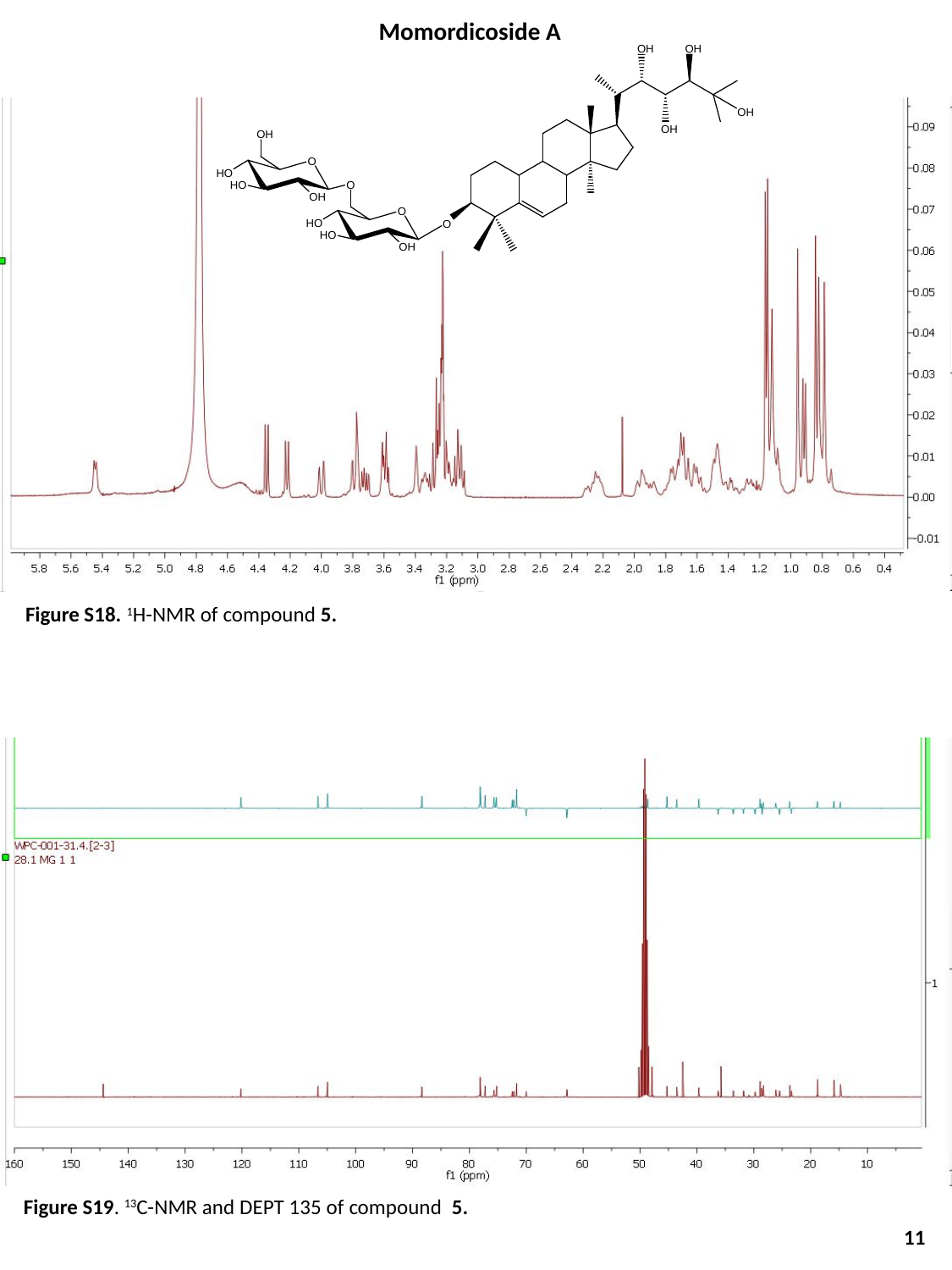

Momordicoside A
Figure S18. 1H-NMR of compound 5.
Figure S19. 13C-NMR and DEPT 135 of compound 5.
11

## Slide 15
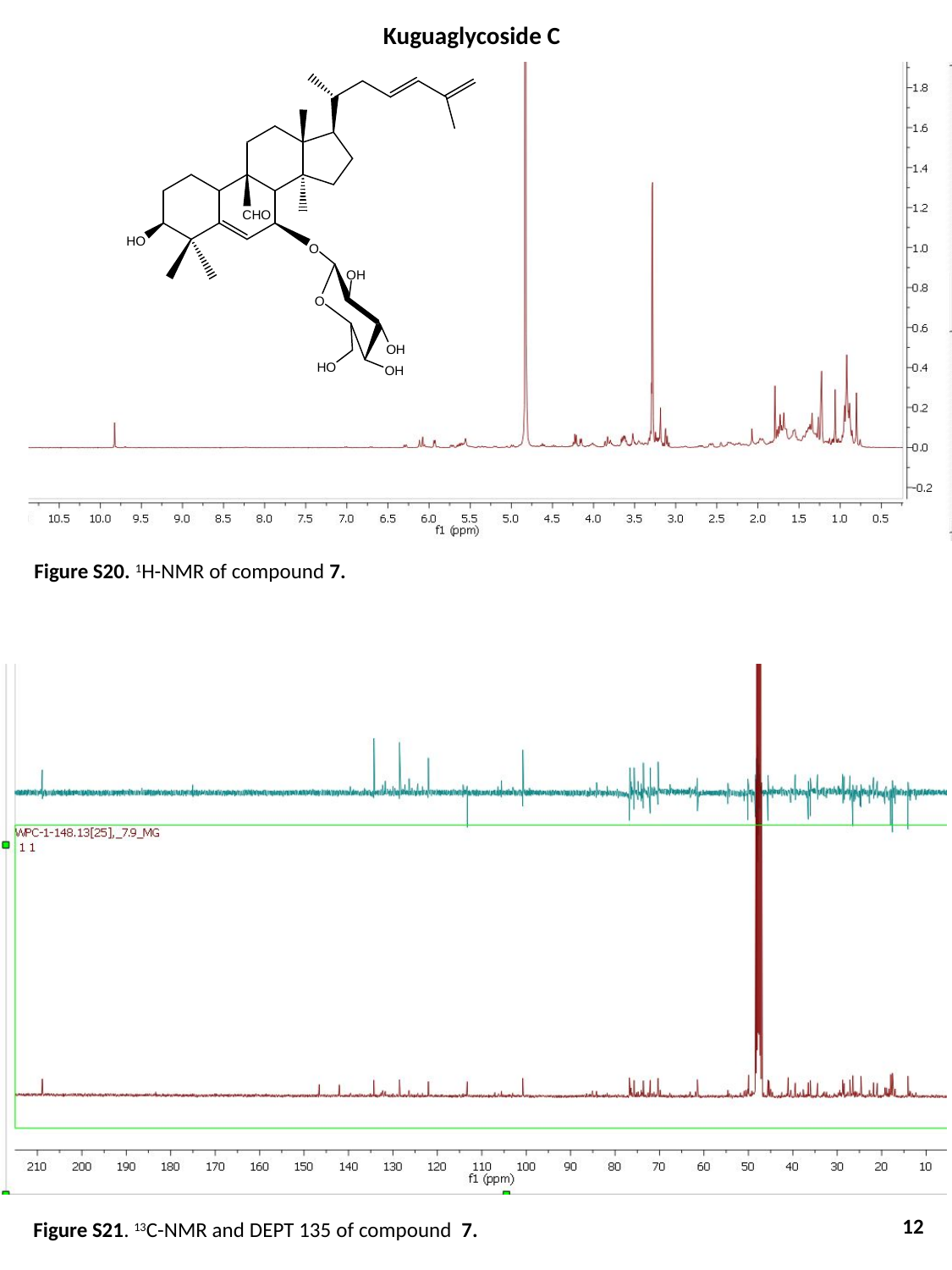

Kuguaglycoside C
Figure S20. 1H-NMR of compound 7.
12
Figure S21. 13C-NMR and DEPT 135 of compound 7.

## Slide 16
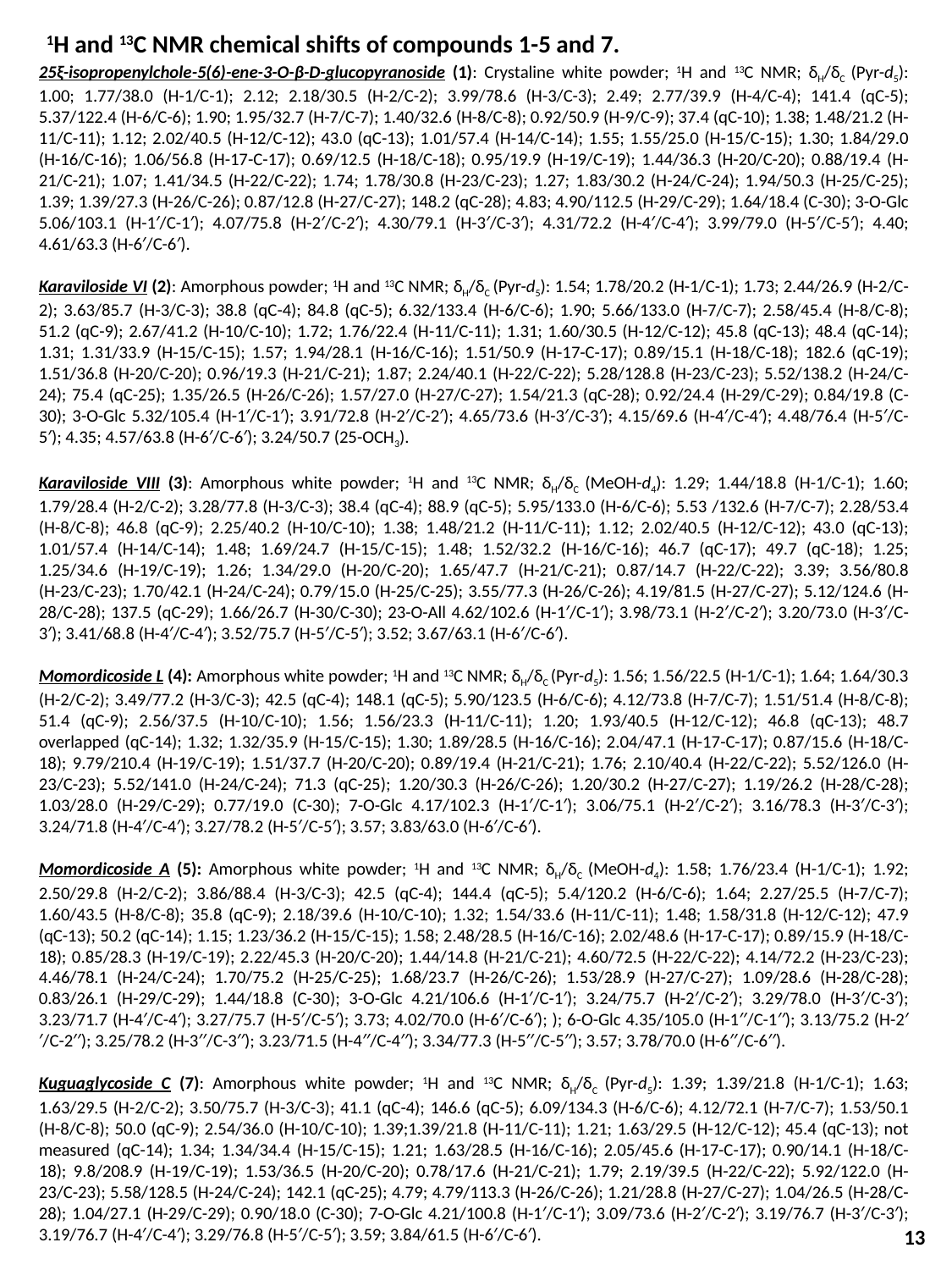

1H and 13C NMR chemical shifts of compounds 1-5 and 7.
25ξ-isopropenylchole-5(6)-ene-3-O-β-D-glucopyranoside (1): Crystaline white powder; 1H and 13C NMR; δH/δC (Pyr-d5): 1.00; 1.77/38.0 (H-1/C-1); 2.12; 2.18/30.5 (H-2/C-2); 3.99/78.6 (H-3/C-3); 2.49; 2.77/39.9 (H-4/C-4); 141.4 (qC-5); 5.37/122.4 (H-6/C-6); 1.90; 1.95/32.7 (H-7/C-7); 1.40/32.6 (H-8/C-8); 0.92/50.9 (H-9/C-9); 37.4 (qC-10); 1.38; 1.48/21.2 (H-11/C-11); 1.12; 2.02/40.5 (H-12/C-12); 43.0 (qC-13); 1.01/57.4 (H-14/C-14); 1.55; 1.55/25.0 (H-15/C-15); 1.30; 1.84/29.0 (H-16/C-16); 1.06/56.8 (H-17-C-17); 0.69/12.5 (H-18/C-18); 0.95/19.9 (H-19/C-19); 1.44/36.3 (H-20/C-20); 0.88/19.4 (H-21/C-21); 1.07; 1.41/34.5 (H-22/C-22); 1.74; 1.78/30.8 (H-23/C-23); 1.27; 1.83/30.2 (H-24/C-24); 1.94/50.3 (H-25/C-25); 1.39; 1.39/27.3 (H-26/C-26); 0.87/12.8 (H-27/C-27); 148.2 (qC-28); 4.83; 4.90/112.5 (H-29/C-29); 1.64/18.4 (C-30); 3-O-Glc 5.06/103.1 (H-1′/C-1′); 4.07/75.8 (H-2′/C-2′); 4.30/79.1 (H-3′/C-3′); 4.31/72.2 (H-4′/C-4′); 3.99/79.0 (H-5′/C-5′); 4.40; 4.61/63.3 (H-6′/C-6′).
Karaviloside VI (2): Amorphous powder; 1H and 13C NMR; δH/δC (Pyr-d5): 1.54; 1.78/20.2 (H-1/C-1); 1.73; 2.44/26.9 (H-2/C-2); 3.63/85.7 (H-3/C-3); 38.8 (qC-4); 84.8 (qC-5); 6.32/133.4 (H-6/C-6); 1.90; 5.66/133.0 (H-7/C-7); 2.58/45.4 (H-8/C-8); 51.2 (qC-9); 2.67/41.2 (H-10/C-10); 1.72; 1.76/22.4 (H-11/C-11); 1.31; 1.60/30.5 (H-12/C-12); 45.8 (qC-13); 48.4 (qC-14); 1.31; 1.31/33.9 (H-15/C-15); 1.57; 1.94/28.1 (H-16/C-16); 1.51/50.9 (H-17-C-17); 0.89/15.1 (H-18/C-18); 182.6 (qC-19); 1.51/36.8 (H-20/C-20); 0.96/19.3 (H-21/C-21); 1.87; 2.24/40.1 (H-22/C-22); 5.28/128.8 (H-23/C-23); 5.52/138.2 (H-24/C-24); 75.4 (qC-25); 1.35/26.5 (H-26/C-26); 1.57/27.0 (H-27/C-27); 1.54/21.3 (qC-28); 0.92/24.4 (H-29/C-29); 0.84/19.8 (C-30); 3-O-Glc 5.32/105.4 (H-1′/C-1′); 3.91/72.8 (H-2′/C-2′); 4.65/73.6 (H-3′/C-3′); 4.15/69.6 (H-4′/C-4′); 4.48/76.4 (H-5′/C-5′); 4.35; 4.57/63.8 (H-6′/C-6′); 3.24/50.7 (25-OCH3).
Karaviloside VIII (3): Amorphous white powder; 1H and 13C NMR; δH/δC (MeOH-d4): 1.29; 1.44/18.8 (H-1/C-1); 1.60; 1.79/28.4 (H-2/C-2); 3.28/77.8 (H-3/C-3); 38.4 (qC-4); 88.9 (qC-5); 5.95/133.0 (H-6/C-6); 5.53 /132.6 (H-7/C-7); 2.28/53.4 (H-8/C-8); 46.8 (qC-9); 2.25/40.2 (H-10/C-10); 1.38; 1.48/21.2 (H-11/C-11); 1.12; 2.02/40.5 (H-12/C-12); 43.0 (qC-13); 1.01/57.4 (H-14/C-14); 1.48; 1.69/24.7 (H-15/C-15); 1.48; 1.52/32.2 (H-16/C-16); 46.7 (qC-17); 49.7 (qC-18); 1.25; 1.25/34.6 (H-19/C-19); 1.26; 1.34/29.0 (H-20/C-20); 1.65/47.7 (H-21/C-21); 0.87/14.7 (H-22/C-22); 3.39; 3.56/80.8 (H-23/C-23); 1.70/42.1 (H-24/C-24); 0.79/15.0 (H-25/C-25); 3.55/77.3 (H-26/C-26); 4.19/81.5 (H-27/C-27); 5.12/124.6 (H-28/C-28); 137.5 (qC-29); 1.66/26.7 (H-30/C-30); 23-O-All 4.62/102.6 (H-1′/C-1′); 3.98/73.1 (H-2′/C-2′); 3.20/73.0 (H-3′/C-3′); 3.41/68.8 (H-4′/C-4′); 3.52/75.7 (H-5′/C-5′); 3.52; 3.67/63.1 (H-6′/C-6′).
Momordicoside L (4): Amorphous white powder; 1H and 13C NMR; δH/δC (Pyr-d5): 1.56; 1.56/22.5 (H-1/C-1); 1.64; 1.64/30.3 (H-2/C-2); 3.49/77.2 (H-3/C-3); 42.5 (qC-4); 148.1 (qC-5); 5.90/123.5 (H-6/C-6); 4.12/73.8 (H-7/C-7); 1.51/51.4 (H-8/C-8); 51.4 (qC-9); 2.56/37.5 (H-10/C-10); 1.56; 1.56/23.3 (H-11/C-11); 1.20; 1.93/40.5 (H-12/C-12); 46.8 (qC-13); 48.7 overlapped (qC-14); 1.32; 1.32/35.9 (H-15/C-15); 1.30; 1.89/28.5 (H-16/C-16); 2.04/47.1 (H-17-C-17); 0.87/15.6 (H-18/C-18); 9.79/210.4 (H-19/C-19); 1.51/37.7 (H-20/C-20); 0.89/19.4 (H-21/C-21); 1.76; 2.10/40.4 (H-22/C-22); 5.52/126.0 (H-23/C-23); 5.52/141.0 (H-24/C-24); 71.3 (qC-25); 1.20/30.3 (H-26/C-26); 1.20/30.2 (H-27/C-27); 1.19/26.2 (H-28/C-28); 1.03/28.0 (H-29/C-29); 0.77/19.0 (C-30); 7-O-Glc 4.17/102.3 (H-1′/C-1′); 3.06/75.1 (H-2′/C-2′); 3.16/78.3 (H-3′/C-3′); 3.24/71.8 (H-4′/C-4′); 3.27/78.2 (H-5′/C-5′); 3.57; 3.83/63.0 (H-6′/C-6′).
Momordicoside A (5): Amorphous white powder; 1H and 13C NMR; δH/δC (MeOH-d4): 1.58; 1.76/23.4 (H-1/C-1); 1.92; 2.50/29.8 (H-2/C-2); 3.86/88.4 (H-3/C-3); 42.5 (qC-4); 144.4 (qC-5); 5.4/120.2 (H-6/C-6); 1.64; 2.27/25.5 (H-7/C-7); 1.60/43.5 (H-8/C-8); 35.8 (qC-9); 2.18/39.6 (H-10/C-10); 1.32; 1.54/33.6 (H-11/C-11); 1.48; 1.58/31.8 (H-12/C-12); 47.9 (qC-13); 50.2 (qC-14); 1.15; 1.23/36.2 (H-15/C-15); 1.58; 2.48/28.5 (H-16/C-16); 2.02/48.6 (H-17-C-17); 0.89/15.9 (H-18/C-18); 0.85/28.3 (H-19/C-19); 2.22/45.3 (H-20/C-20); 1.44/14.8 (H-21/C-21); 4.60/72.5 (H-22/C-22); 4.14/72.2 (H-23/C-23); 4.46/78.1 (H-24/C-24); 1.70/75.2 (H-25/C-25); 1.68/23.7 (H-26/C-26); 1.53/28.9 (H-27/C-27); 1.09/28.6 (H-28/C-28); 0.83/26.1 (H-29/C-29); 1.44/18.8 (C-30); 3-O-Glc 4.21/106.6 (H-1′/C-1′); 3.24/75.7 (H-2′/C-2′); 3.29/78.0 (H-3′/C-3′); 3.23/71.7 (H-4′/C-4′); 3.27/75.7 (H-5′/C-5′); 3.73; 4.02/70.0 (H-6′/C-6′); ); 6-O-Glc 4.35/105.0 (H-1′′/C-1′′); 3.13/75.2 (H-2′′/C-2′′); 3.25/78.2 (H-3′′/C-3′′); 3.23/71.5 (H-4′′/C-4′′); 3.34/77.3 (H-5′′/C-5′′); 3.57; 3.78/70.0 (H-6′′/C-6′′).
Kuguaglycoside C (7): Amorphous white powder; 1H and 13C NMR; δH/δC (Pyr-d5): 1.39; 1.39/21.8 (H-1/C-1); 1.63; 1.63/29.5 (H-2/C-2); 3.50/75.7 (H-3/C-3); 41.1 (qC-4); 146.6 (qC-5); 6.09/134.3 (H-6/C-6); 4.12/72.1 (H-7/C-7); 1.53/50.1 (H-8/C-8); 50.0 (qC-9); 2.54/36.0 (H-10/C-10); 1.39;1.39/21.8 (H-11/C-11); 1.21; 1.63/29.5 (H-12/C-12); 45.4 (qC-13); not measured (qC-14); 1.34; 1.34/34.4 (H-15/C-15); 1.21; 1.63/28.5 (H-16/C-16); 2.05/45.6 (H-17-C-17); 0.90/14.1 (H-18/C-18); 9.8/208.9 (H-19/C-19); 1.53/36.5 (H-20/C-20); 0.78/17.6 (H-21/C-21); 1.79; 2.19/39.5 (H-22/C-22); 5.92/122.0 (H-23/C-23); 5.58/128.5 (H-24/C-24); 142.1 (qC-25); 4.79; 4.79/113.3 (H-26/C-26); 1.21/28.8 (H-27/C-27); 1.04/26.5 (H-28/C-28); 1.04/27.1 (H-29/C-29); 0.90/18.0 (C-30); 7-O-Glc 4.21/100.8 (H-1′/C-1′); 3.09/73.6 (H-2′/C-2′); 3.19/76.7 (H-3′/C-3′); 3.19/76.7 (H-4′/C-4′); 3.29/76.8 (H-5′/C-5′); 3.59; 3.84/61.5 (H-6′/C-6′).
13

## Slide 17
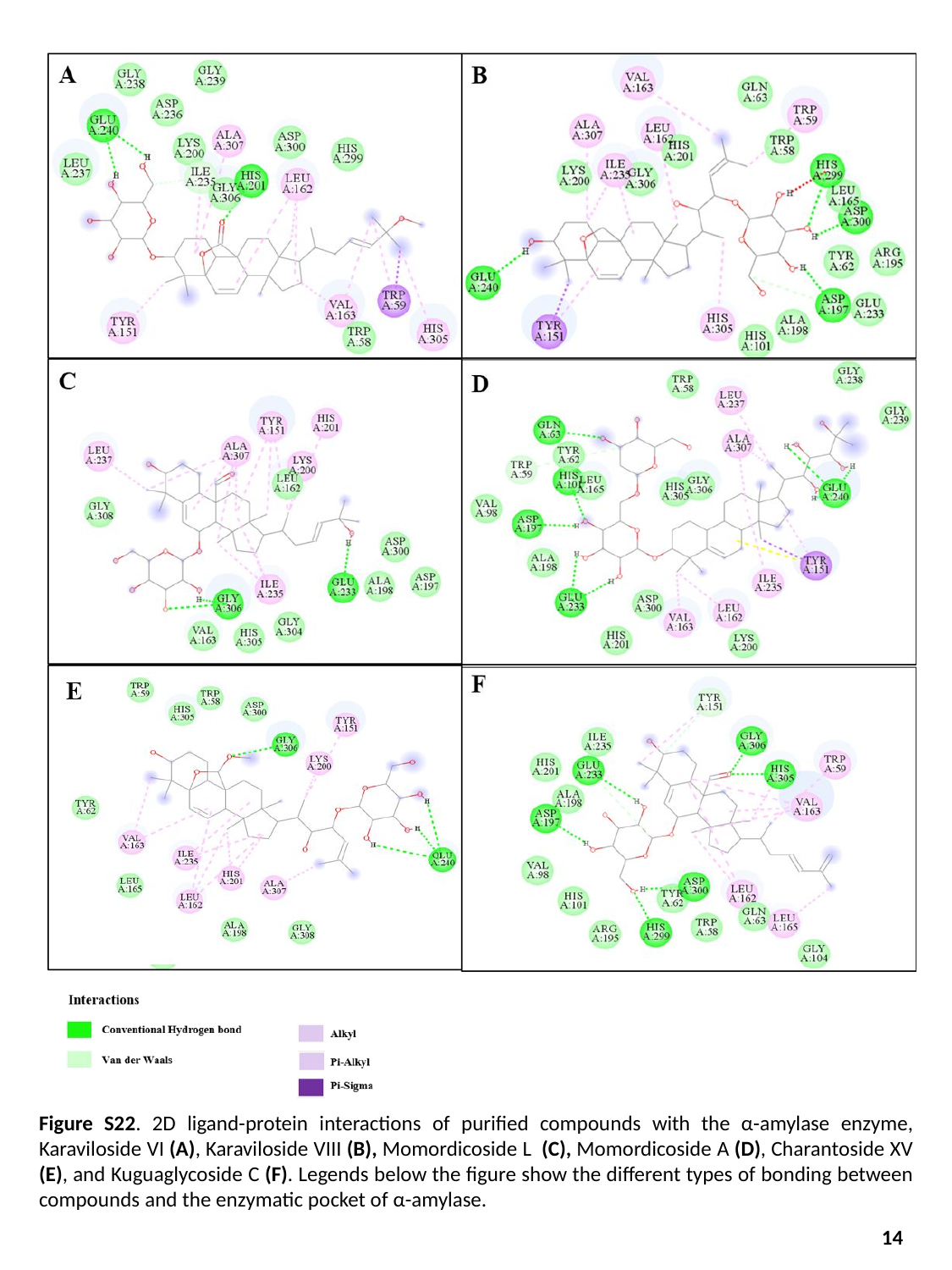

Figure S22. 2D ligand-protein interactions of purified compounds with the α-amylase enzyme, Karaviloside VI (A), Karaviloside VIII (B), Momordicoside L (C), Momordicoside A (D), Charantoside XV (E), and Kuguaglycoside C (F). Legends below the figure show the different types of bonding between compounds and the enzymatic pocket of α-amylase.
14

## Slide 18
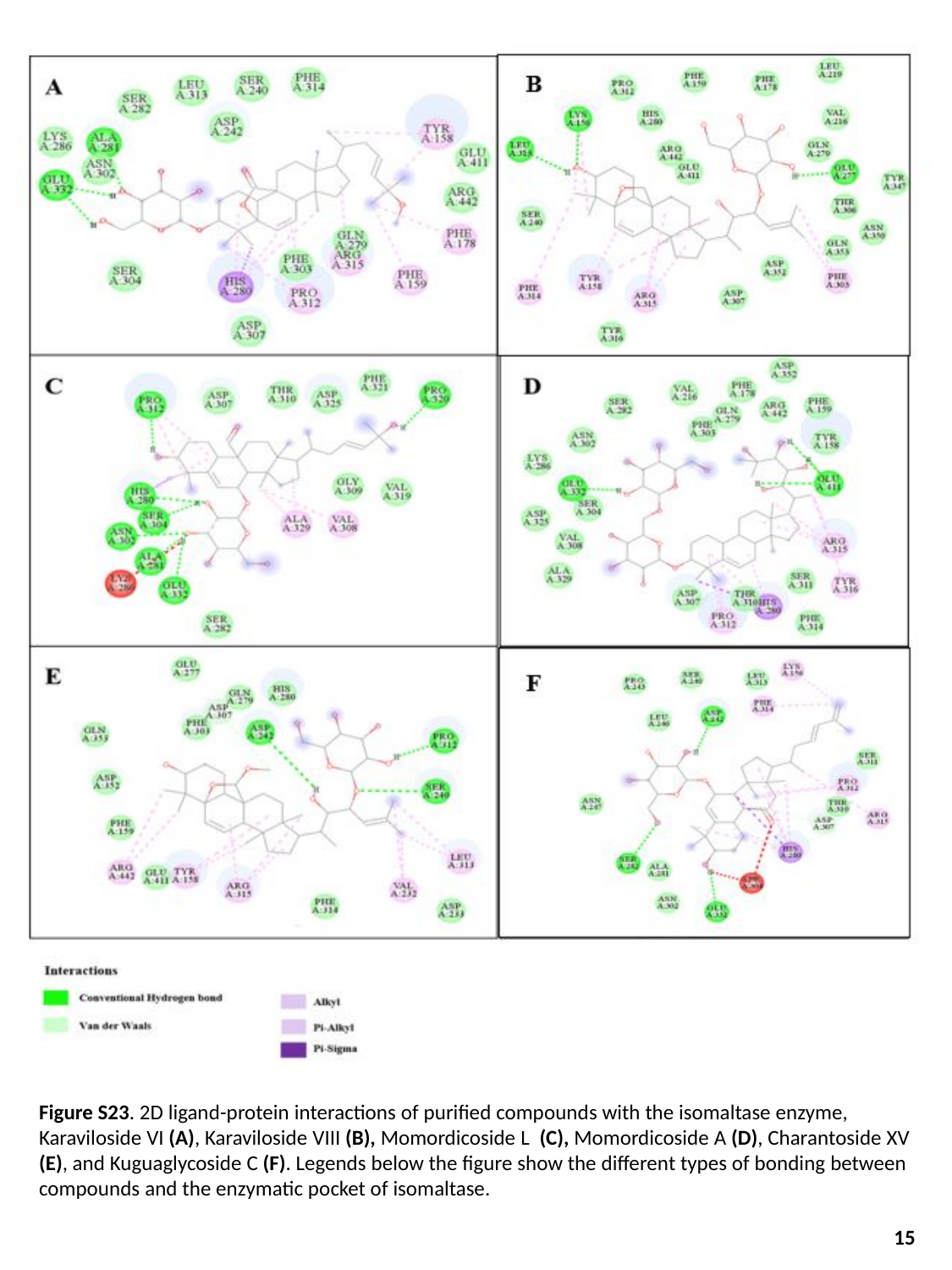

Figure S23. 2D ligand-protein interactions of purified compounds with the isomaltase enzyme, Karaviloside VI (A), Karaviloside VIII (B), Momordicoside L (C), Momordicoside A (D), Charantoside XV (E), and Kuguaglycoside C (F). Legends below the figure show the different types of bonding between compounds and the enzymatic pocket of isomaltase.
15

## Slide 19
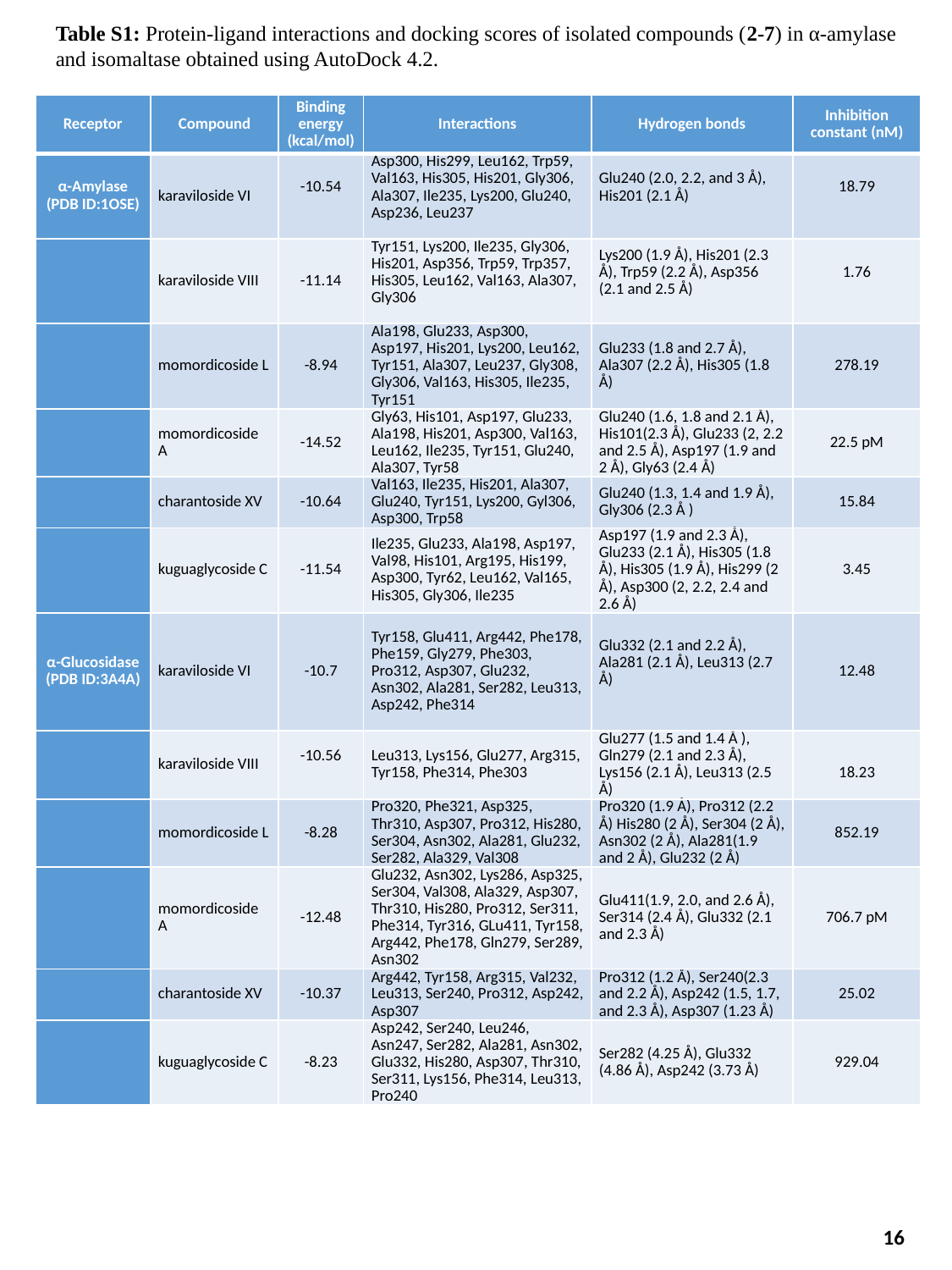

Table S1: Protein-ligand interactions and docking scores of isolated compounds (2-7) in α-amylase and isomaltase obtained using AutoDock 4.2.
| Receptor | Compound | Binding energy (kcal/mol) | Interactions | Hydrogen bonds | Inhibition constant (nM) |
| --- | --- | --- | --- | --- | --- |
| α-Amylase (PDB ID:1OSE) | karaviloside VI | -10.54 | Asp300, His299, Leu162, Trp59, Val163, His305, His201, Gly306, Ala307, Ile235, Lys200, Glu240, Asp236, Leu237 | Glu240 (2.0, 2.2, and 3 Å), His201 (2.1 Å) | 18.79 |
| | karaviloside VIII | -11.14 | Tyr151, Lys200, Ile235, Gly306, His201, Asp356, Trp59, Trp357, His305, Leu162, Val163, Ala307, Gly306 | Lys200 (1.9 Å), His201 (2.3 Å), Trp59 (2.2 Å), Asp356 (2.1 and 2.5 Å) | 1.76 |
| | momordicoside L | -8.94 | Ala198, Glu233, Asp300, Asp197, His201, Lys200, Leu162, Tyr151, Ala307, Leu237, Gly308, Gly306, Val163, His305, Ile235, Tyr151 | Glu233 (1.8 and 2.7 Å), Ala307 (2.2 Å), His305 (1.8 Å) | 278.19 |
| | momordicoside A | -14.52 | Gly63, His101, Asp197, Glu233, Ala198, His201, Asp300, Val163, Leu162, Ile235, Tyr151, Glu240, Ala307, Tyr58 | Glu240 (1.6, 1.8 and 2.1 Å), His101(2.3 Å), Glu233 (2, 2.2 and 2.5 Å), Asp197 (1.9 and 2 Å), Gly63 (2.4 Å) | 22.5 pM |
| | charantoside XV | -10.64 | Val163, Ile235, His201, Ala307, Glu240, Tyr151, Lys200, Gyl306, Asp300, Trp58 | Glu240 (1.3, 1.4 and 1.9 Å), Gly306 (2.3 Å ) | 15.84 |
| | kuguaglycoside C | -11.54 | Ile235, Glu233, Ala198, Asp197, Val98, His101, Arg195, His199, Asp300, Tyr62, Leu162, Val165, His305, Gly306, Ile235 | Asp197 (1.9 and 2.3 Å), Glu233 (2.1 Å), His305 (1.8 Å), His305 (1.9 Å), His299 (2 Å), Asp300 (2, 2.2, 2.4 and 2.6 Å) | 3.45 |
| α-Glucosidase (PDB ID:3A4A) | karaviloside VI | -10.7 | Tyr158, Glu411, Arg442, Phe178, Phe159, Gly279, Phe303, Pro312, Asp307, Glu232, Asn302, Ala281, Ser282, Leu313, Asp242, Phe314 | Glu332 (2.1 and 2.2 Å), Ala281 (2.1 Å), Leu313 (2.7 Å) | 12.48 |
| | karaviloside VIII | -10.56 | Leu313, Lys156, Glu277, Arg315, Tyr158, Phe314, Phe303 | Glu277 (1.5 and 1.4 Å ), Gln279 (2.1 and 2.3 Å), Lys156 (2.1 Å), Leu313 (2.5 Å) | 18.23 |
| | momordicoside L | -8.28 | Pro320, Phe321, Asp325, Thr310, Asp307, Pro312, His280, Ser304, Asn302, Ala281, Glu232, Ser282, Ala329, Val308 | Pro320 (1.9 Å), Pro312 (2.2 Å) His280 (2 Å), Ser304 (2 Å), Asn302 (2 Å), Ala281(1.9 and 2 Å), Glu232 (2 Å) | 852.19 |
| | momordicoside A | -12.48 | Glu232, Asn302, Lys286, Asp325, Ser304, Val308, Ala329, Asp307, Thr310, His280, Pro312, Ser311, Phe314, Tyr316, GLu411, Tyr158, Arg442, Phe178, Gln279, Ser289, Asn302 | Glu411(1.9, 2.0, and 2.6 Å), Ser314 (2.4 Å), Glu332 (2.1 and 2.3 Å) | 706.7 pM |
| | charantoside XV | -10.37 | Arg442, Tyr158, Arg315, Val232, Leu313, Ser240, Pro312, Asp242, Asp307 | Pro312 (1.2 Å), Ser240(2.3 and 2.2 Å), Asp242 (1.5, 1.7, and 2.3 Å), Asp307 (1.23 Å) | 25.02 |
| | kuguaglycoside C | -8.23 | Asp242, Ser240, Leu246, Asn247, Ser282, Ala281, Asn302, Glu332, His280, Asp307, Thr310, Ser311, Lys156, Phe314, Leu313, Pro240 | Ser282 (4.25 Å), Glu332 (4.86 Å), Asp242 (3.73 Å) | 929.04 |
16
